# Improved Test Fixture for Collecting Microcontact Performance and Reliability Data

**DOI:** 10.3390/mi15050597

**Published:** 2024-04-29

**Authors:** Turja Nandy, Ronald A. Coutu, Rafee Mahbub

**Affiliations:** Department of Electrical and Computer Engineering, Marquette University, Milwaukee, WI, 53233, USA; turja.nandy@marquette.edu (T.N.); rafee.mahbub@marquette.edu (R.M.)

**Keywords:** microswitch, microcontact, MEMS fabrication, reliability, contact resistance

## Abstract

Microelectromechanical systems (MEMS) ohmic contact switches are considered to be a promising candidate for wireless communication applications. The longevity of MEMS switches is directly related to the reliability and performance of microcontacts. In this work, an improved microcontact test fixture with high actuation rates (KHz) and highly precise position control (nm) and force (nN) control was developed. Here, we collected microcontact performance data from initial contact tests (ICT) and microcontact reliability data from cold switched tests (CST). To perform these tests with our test fixture, we fabricated MEMS microcontact test structures with relatively high Young’s modulus electroplated Nickel (Ni)-based, fixed–fixed beam structure with Au/RuO_2_ bimetallic microcontacts. These structures were characterized for forces ranging from 200–1000 µN in ICT tests. In a CST test, the tested microcontact survived more than 200 million cycles at a 1 KHz cycle rate, with a stable contact resistance value ranging between 3.8–5.2 Ω. These experiments validate the potentiality of our microcontact test fixture, and will facilitate further investigation on advanced microcontacts to enhance the MEMS switch’s reliability.

## 1. Introduction

Microelectromechanical systems (MEMS) devices are miniaturized devices with integrated micron-sized electrical and mechanical components. MEMS metal contact microswitches are suitable for various applications such as cell phones, 5G/6G networks, phased array antennas, radars, and internet of things (IOT) devices. MEMS microswitches provide low contact resistance with low power consumption [1,2,3]. The switching operation is governed by the ohmic contact, achieved through physical contact between the lower and upper contact regions. Thus, the reliability of microcontacts drives a MEMS switch’s performance and longevity. Researchers indicated contact failure in MEMS switches due to material transfer [4], adhesion [5,6], molten metal bridge formation [7,8], contamination [9], and contact bounce [10].

Understanding the failure mechanisms requires sophisticated experimental test fixtures that are capable of real-time data acquisition with precise control over millions of cycles. Studies were carried out using a nanoindenter, scanning probe microscope (SPM), and an atomic force microscope (AFM) to investigate the MEMS switch’s reliability performance. In AFM and SPM based test fixtures, a fixed–fixed beam structure with a laser light was employed to track the development of contact force in the microcontact area [11,12,13]. Compared to the AFM- and SPM-based test setups, the primary benefit of the nanoindenter-based test configuration was its direct ability to quantify contact force [14,15]. However, their inability to perform real-time measurements of contact resistance with variable cycle rates and contact forces, and susceptibility to atmospheric contaminations, are limiting factors in AFM, SPM, and nanoindentation techniques. Researchers developed text fixtures to address the contamination issue, but are limited by cycle rate and postmortem analysis [16]. Another test fixture was assembled for overcoming the above issues. But this test fixture faced many hardware and software issues, and ultimately, Mahanta et al. failed to collect any usable experimental data to validate the fixture’s suitability for testing microcontacts or microswitches [17]. In addition, gold (Au) is the most common microcontact material. But it suffers from lifetime issues and is very prone to contact degradation due to its low hardness [18]. Ruthenium oxide (RuO_2_) is one of the promising materials because of its material property [19,20,21]. To overcome the contact failure issues with gold (Au) microcontact, it was reported that relatively harder ruthenium oxide (RuO_2_) has encouraging results to be a reliable microcontact material [22]. But significant research is required to prove its reliability and performance in MEMS switches.

In this paper, we presented an improved automated microcontact test fixture capable of real-time contact resistance measurement with high precision, coupled with variable high cycle and high actuation rates. We fabricated a fixed-fixed Ni beam-based microcontact test structure following microfabrication techniques. Using this structure in our microcontact test fixture, we collected precision force data, beam restoring force data, initial contact test data (ICT), and cold switched testing (CST) data for Au/RuO_2_ microcontacts to validate the test fixture’s suitability. These collected data will help in future research work investigating advanced microcontacts’ performance.

## 2. Improved Microcontact Test Fixture Assembly

### 2.1. Test Fixture Design

**Figure 1:**
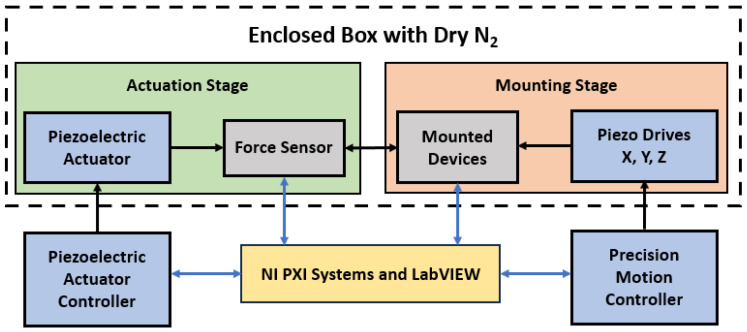
Block diagram for the experimental test setup.

**Figure 2:**
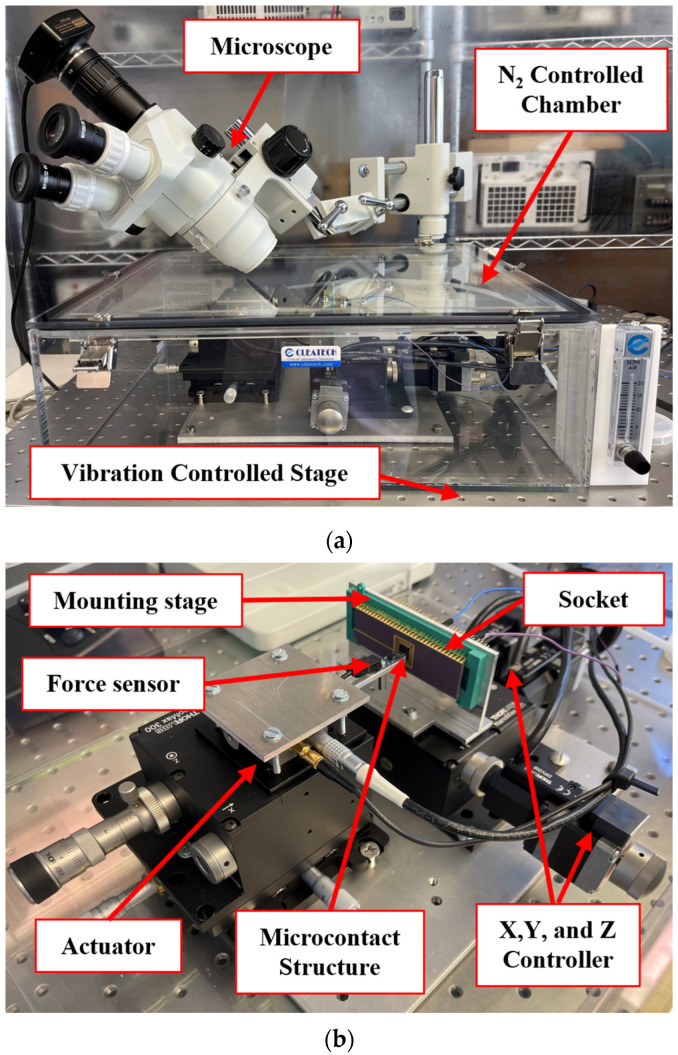
(**a**) Experimental test fixture, and (**b**) different components of the experimental setup.

We used our unique and improved automated test fixture to collect microcontact performance and reliability data. Figure 1 presents the block diagram of our test fixture, and Figure 2a shows the pictorial image of the experimental fixture with microscope. The test assembly is placed in a N_2_ controlled enclosed dry box to reduce contamination. This assembly is divided into two main parts: the mounting stage for the device, and the actuation stage for the force sensor. The mounting stage is a NanoMax300 (ThorLabs, Inc., Newton, NJ, USA) stage that holds the socket (device holder) where the microcontact structure is placed and the wire is bonded; see Figure 2b. This mounting stage has three DRV108 (ThorLabs, Inc., Newton, NJ, USA) piezo drives which allows the stage to move in all three directions (x, y, and z). The stage can travel up to 8 mm with step size ranges between 1 mm to 1 nm, and it is controlled using a precision motion controller.

The actuation stage consists of a force sensor, a piezoelectric actuator, and three high precision differential micrometers. The force sensor is FT-S10000 (FemtoTools AG, Buchs, Switzerland) which can provide up to 10,000 µN force with nN force resolution (with a gain 4900–5000 µN/V and tip area 50 × 50 µm^2^). When the mounting stage is fixed, the PAZ005 (ThorLabs, Inc., Newton, NJ, USA) piezoelectric actuator allows the force sensor to move towards the microcontact structure. The force sensor can move up to 20 µm with step size ranging between 1 nm to 20 µm. This piezoelectric actuator is controlled using a piezo controller. Moreover, the actuation stage can be manually moved by using precision differential micrometers while needed. Both controllers, force sensor, and mounted microcontact structures are connected to a PXIe 1062Q (National Instruments, Austin, TX, USA). The complete fixture is operated via novel LabVIEW (National Instruments, Austin, TX, USA) programs and the measurements are collected using programmable power supply, digital multimeter, and function generator of an PXI (National Instruments, Austin, TX, USA) system. Figure 2a shows all the components of the experimental setup, and Figure 2b shows a force sensor placed on a microcontact structure to actuate it, making the system is ready for measurement.

### 2.2. Alignment between Force Sensor Tip and Microcontact Structure

**Figure 3:**
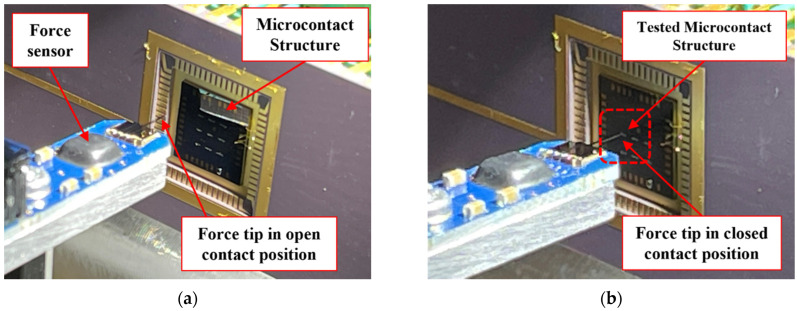
Alignment between force sensor tip and microcontact structure in our test fixture. The force sensor tip in (**a**) open contact position, and (**b**) close contact position.

When the microcontact structure is placed in the test fixture, force sensor and microcontact structure were aligned to start the testing procedure. Firstly, the force sensor was initialized. As the microcontact structure was placed in mounting stage, the microcontact structure was taken in front of force sensor tip controlling X, Y, and Z piezo drives connected with the mounting stage. This travel minimizes the gap between the force sensor and the desired microcontact structure from mm range to µm range. Then, the force sensor tip was taken towards the desired microcontact structure using a piezoelectric actuator and high-precision differential micrometers connected with the actuation stage. Figure 3 shows the travel and alignment process between force sensor and microcontact structure.

## 3. Design and Fabrication of Microcontact Test Structure

### 3.1. Beam Modeling for Microcontact Test Structure

**Figure 4:**
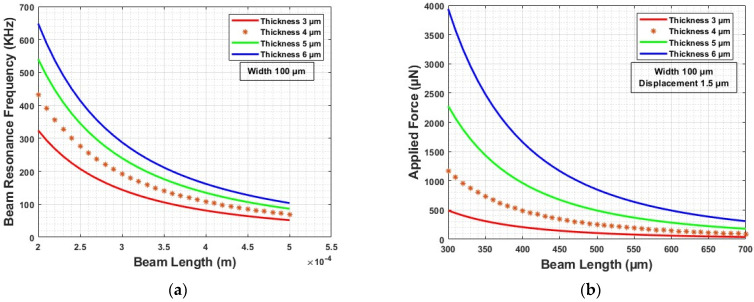
(**a**) Beam resonance frequency modeling, and (**b**) beam displacement modeling.

Our microcontact test structure mimics the principle of a fixed–fixed beam geometry. We chose electroplated Nickel (Ni) beam because of its hardness and good yield strength. The stiffness ($K_{fixed\text{–}fixed}$) of beam is a function of the Young’s modulus ($E_{Ni}$) and is directly related to the length, width, and thickness ($L_{beam}$, $W_{beam}$ and $T_{beam}$). Based on the stiffness and density of Ni ($\rho_{Ni}$), the resonance frequency ($f_{resonance}$) can be calculated. Resonance frequency determines the highest actuation frequency ($f_{actuation}$) [23,24]. Figure 4a shows beam resonance frequency variation. Based on the modeling and analysis, Ni beam with 500 µm length, 100 µm width, and 4–5 µm thickness were chosen to design our microcontact test structure. The resonance frequency ranges between 60–80 KHz. So, the actuation frequency should be under this value.

(1) $$K_{fixed\text{–}fixed}=\frac{16E_{Ni}W_{beam}{\left(T_{beam}\right)}^{3}}{{\left(L_{beam}\right)}^{3}}$$

(2) $$f_{resonant}=\frac{1}{2\pi }\sqrt{\frac{2K_{fixed\text{–}fixed}}{L_{beam}W_{beam}T_{beam}\rho_{Ni}}}$$

(3) $${f_{actuation}<f}_{resonance}$$

Based on our beam parameters, we analyzed the maximum force needed to achieve our desired displacement of 1.5 µm. Equation (4) shows that beam displacement is a function of applied force ($F_{applied}$), beam dimensions and Young’s modulus. From Figure 4b, we found that our maximum force does not exceed 500 µN. We considered the minimum yield strength of Ni ($Y_{Ni}$) and maximum applied force to determine the anchor area, and an anchor area of 100 × 100 µm^2^ should be enough to hold the beam. In addition, when the beam is in equilibrium, the applied force is equal to the mechanical beam restoring force ($F_{restoring}$), which can be found from stiffness and maximum displacement (shown in Equation (5)) [15,25]. The calculated beam-restoring force is 250 µN for 500 µm long beam.

(4) $$F_{applied}=\frac{16E_{Ni}d_{max}W_{beam}{\left(T_{beam}\right)}^{3}}{{\left(L_{beam}\right)}^{3}}$$

(5) $${F_{restoring}=K}_{fixed\text{–}fixed}d_{max}$$

(6) $$Anchorarea>\frac{MaximumAppliedForce}{Y_{Ni}}$$

### 3.2. Microcontact Test Structure Fabrication

**Figure 5:**
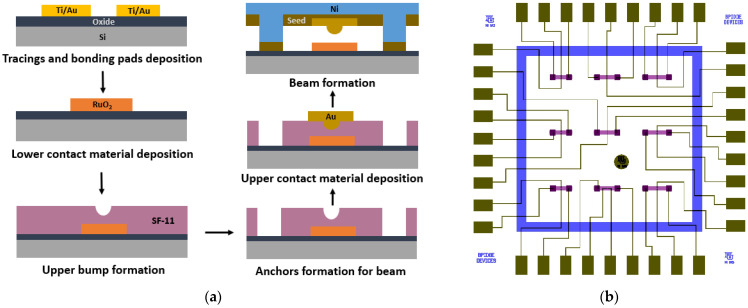
(**a**) Process flow of microcontact test structure, and (**b**) die level mask layout.

The bottom-up MEMS fabrication process method was followed to fabricate the advanced microcontact test structure. A 450 µm ± 20-µm-thick single-sided polished prime silicon (Si) wafer was used as a substrate. Figure 5a shows the process flow of our advanced microcontact structure fabrication. The glass mask set was designed in L-Edit and fabricated using lithography and wet etching techniques. Figure 5b shows the die-level mask layout. Every step of our fabrication process is briefly discussed in this section.

**Figure 6:**
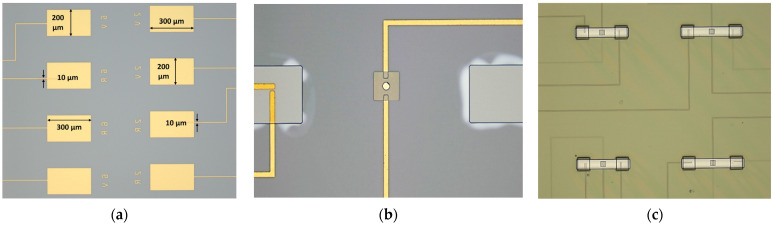
Optical microscopic image of (**a**) fabricated tracing lines and bonding pads, (**b**) fabricated lower contact material, upper bump, upper contact material, and anchors, and (**c**) electroplated Ni beams.

The fabrication process for the microcontact test structure is thoroughly discussed in this Section. Firstly, a thin insulation layer of SiO_2_ was deposited before doing further processing. Then, tracing line (10 µm wide) and bonding pad (300 × 200 µm^2^) features were patterned using 1.8 µm thick S1818 PR (Kayaku Advanced Materials, Inc., Westborough, MA, USA). PR was coated at 4000 rpm for 45 s, baked at 110 °C for 60 s, exposed to UV light for 5.5 s, and finally developed with 351: DIW [1:5] developer for 35 s. Next, the wafer with developed patterns was placed into the dual deposition system for metal deposition. A 30 nm Titanium (Ti) adhesion film followed by a 350 nm Gold (Au) thin film was performed using the e-beam evaporation technique. A metal lift-off process was performed with 1165 PR stripper to remove the excess deposited metal from the undesired locations by placing it into the ultrasonic bath chamber for 10 min. Figure 6a demonstrates microscopic image of deposited metal features for tracing lines and bonding pads. To deposit the lower contact material on the 60 × 60 µm^2^ lower contact region, S1818 PR was coated, aligned, exposed, and developed. Then, 150 nm RuO_2_ was deposited using reactive sputter deposition technique, with 75 W RF power, Ar:O_2_ [18:0.5] gas flow and 5.5 mTorr chamber pressure for an hour. A traditional lift-off process was conducted to get the final features. Figure 6b shows the lower contact material structure.

Upper contact bump and beam anchor processing required using two photoresists, SF-11 and S1818. Thick SF-11 polymer (Kayaku Advanced Materials, Inc., Westborough, MA, USA) was spin-coated on a wafer with 4000 rpm speed at 2000 rpm/s ramp rate for 45 s, and then baked at 200 °C for 2 min. This process was repeated to get a second layer of SF-11 (total thickness ~2.5 µm). Next, S1818 PR was patterned on top of SF-11 layer. Using this patterned S1818 as a mask, SF-11 was partially exposed with a deep UV (DUV) system (exposure wavelength and time were 220 nm and 60 s). After developing SF-11 with 101A developer for 60 s, followed by cleaning of S1818 and thermal reflow of SF-11, the desired hemispherical upper contact bump was formed. For the anchor area, again, S1818 was patterned for a masking layer on SF-11. Then, the SF-11 was fully exposed to DUV light for 400 s and developed for 80 s. S1818 was cleaned and the anchor area was inspected in profilometer and optical microscope. Figure 6b shows the upper contact bump and processed anchor areas for the beam. Afterwards, S1818 was patterned to deposit upper contact material. Here, we deposited 150 nm Au as the upper contact material using the DC sputter deposition techniques and metal lift-off process. Figure 6b shows the upper contact material deposited on top of the upper contact bump.

Finally, beam processing started with depositing 250 nm chromium (Cr) on prepared SF-11 using the RF sputter technique. The Cr/Au layer acted as a seed layer for the nickel (Ni) electroplating. After the deposition, S1818 film was patterned for beam architecture. The wafer was then placed in electroplating chamber. A 5-µm-thick Ni beam was electroplated using 2.5 V at 60 °C for 3 min. Then, the S1818 was cleaned, and the wafer was placed into the chromium and gold etchant, respectively, to etch 250 nm Cr/Au seed layer. During the Cr and Au etch, the wafer was periodically inspected after every 10 s during the wet metal etch to reduce the selectivity towards Ni. Figure 6c shows microscopic image of electroplated Ni beams. Upon etching the seed layer, the wafer was diced into individual 7.5 × 7.5 mm die using dicing saw. Individual die was soaked into 1165 at 90 °C for 45 min followed by soaking into IPA for 60 s. The die was transferred into a Methanol filled holder and placed into the CO_2_ dryer. The release process was performed for 90 min. After the release process, the die was attached to the socket, and wedge–wire bonding was performed between the bonding pads and socket pads. When the assembly process was finished, the microcontact test structures were ready to be tested.

## 4. Test Results Collected Using Our Test Fixture

### 4.1. Performance Study of Beam and Force Sensor

**Figure 7:**
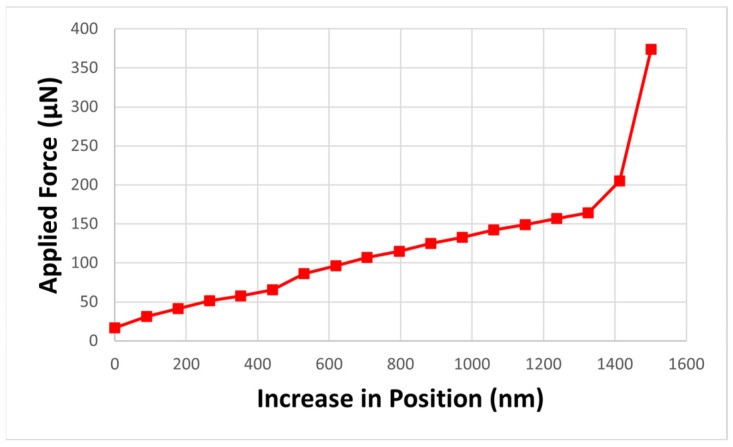
Applied force values according to the position increment.

**Figure 8:**
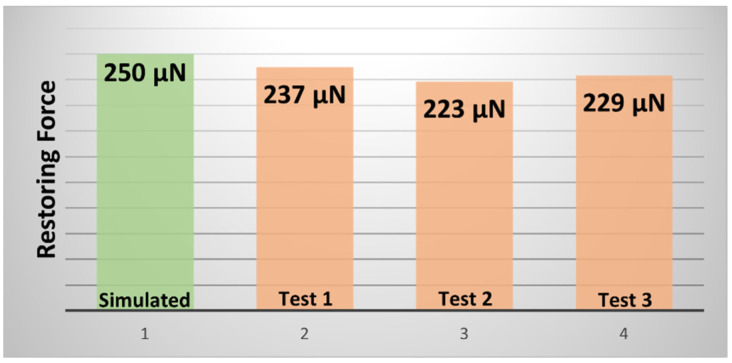
Comparison between simulation and experimental restoring force for the 500 µm long beam.

Firstly, manual alignment of our experimental setup helps us to take the force sensor near the top of the beam. After traveling a certain distance, the integrated force sensor was initialized and was gradually advanced in 10 nm increments until the sensor tip made contact with the beam. We recorded the force values according to the tip position, until the beam touched the bottom contact. Figure 7 shows the force variation according to the position increment. As the position was increased (0–1500 nm), a gradual increase in applied force was observed. These data help us to understand the force sensor behavior, as well as evaluate the force needed to actuate the beam. While gradually taking the applied load away from the beam, the restoring force was measured several times. From three tests with 500 µm beam, 223–237 µN-restoring force was observed. Figure 8 presents a comparison between the simulated and experiment restoring force results. However, the simulated result overpredicts the restoring force. During the beam fabrication step, the sidewalls were etched 5–10 µm. Thus, the width became smaller (~90 µm) than expected (100 µm), and caused the reduction of the restoring force values, which is verified from the beam modeling.

### 4.2. Contact Resistance Modeling

To achieve outstanding microswitching performance, stability and low contact resistance are critical [26,27]. High and erratic contact resistance is produced by low contact force. Plastic deformation of contact asperities occurs under high contact forces, resulting in a reduction of contact resistance [28]. Asperity peaks, or “a-spots”, form the conducting path in microcontacts and have a significant influence on contact resistance [29]. Electrical current can only pass via “a-spots” made during switch closure, which causes constriction resistance because of this diffusive electron transport [30]. Maxwellian spreading resistance theory can be used to analytically explain constriction resistance. In the relatively higher contact force region, plastic deformation happens. That is why contact film contamination takes place, which plays a significant role and needs to be considered in contact resistance calculation [31,32]. In the plastic region, constriction resistance ($R_{constriction}$) can be determined based on contact force ($F_{contact}$), effective contact area ($A_{eff}$), resistivity (ρ), and hardness (H) of the contact material (shown in Equation (7)). Based on constriction resistance and contaminant film resistance ($R_{cf}$), contact resistance ($R_{contact}$) can be calculated using Equation (9) [18,33]. For our test method, the contact resistance is modeled as Equation (9), where beam resistance ($R_{beam}$), sheet resistance ($R_{sheet}$), and parasitic resistance due to electrical connections ($R_{parasitic}$) are subtracted from the measured resistance ($R_{measured}$).

(7) $$R_{constriction}=\frac{\rho }{2}\sqrt{\frac{\pi }{A_{eff}}}=\frac{\rho }{2}\sqrt{\frac{\pi \cdot H}{F_{contact}}}$$

(8) $$R_{contact}=R_{constriction}+R_{cf}$$

(9) $$R_{contact}=R_{measured}-R_{beam}-R_{sheet}-R_{parasitic}$$

### 4.3. Initial Contact Testing (ICT) for Microcontact

**Figure 9:**
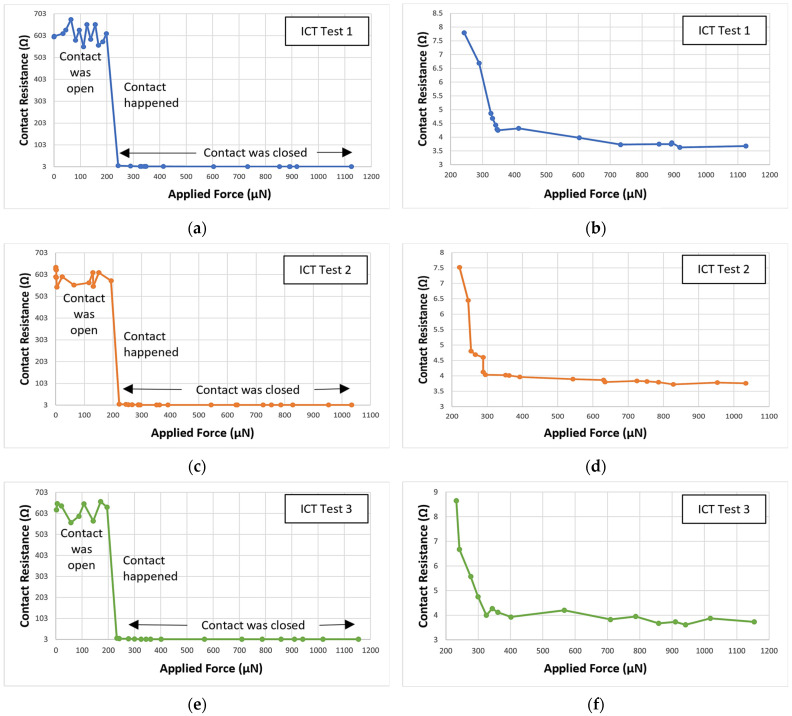
Contact resistance vs. applied force results for three initial contact test (ICT)—(**a**,**c**,**e**) presents full ICT test period, and (**b**,**d**,**f**) presents the contact resistance when contact was fully closed.

**Figure 10:**
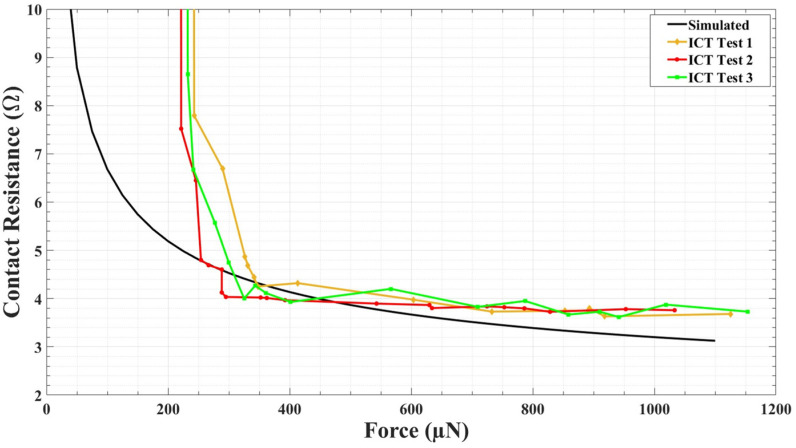
Comparison between simulated and experimental initial contact test (ICT) results.

For the ICT, a DC load was added to the beam. Until the voltage drop across the contact and the current through were detected, we kept moving the force sensor forward. After performing a few ICT, the first contact resistance was recorded when the applied force was approximately 220–250 µN. This position indicates physical contact between upper and lower contact. Here, the applied force includes the initial beam restoring force, i.e., the force required to push the upper contact towards the lower contact until the contact was made. When the contact was made, it was considered to be a zero-contact force point. The contact force can be calculated by subtracting the beam-restoring force from the applied force. Figure 9 shows the ICT results. When the applied force increases from 200 µN to 1000 µN, the contact resistance decreased from approximately 9 Ω to approximately 3.5–5 Ω. In Figure 9a,c,e, the applied force from 0–1200 µN and corresponding contact resistance measurement is presented. These Figures include the contact resistance measurement when the contact was open, when the contact happened, and when the contact was fully closed. In Figure 9b,d,f, the data present the contact resistance measurement only when the contact was fully closed (zoomed images of respective arrowed areas from Figure 9a,c,e). Figure 10 presents the comparison between the simulated and experimental ICT results.

### 4.4. Cold Switched Testing (CST) for Microcontact

**Figure 11:**
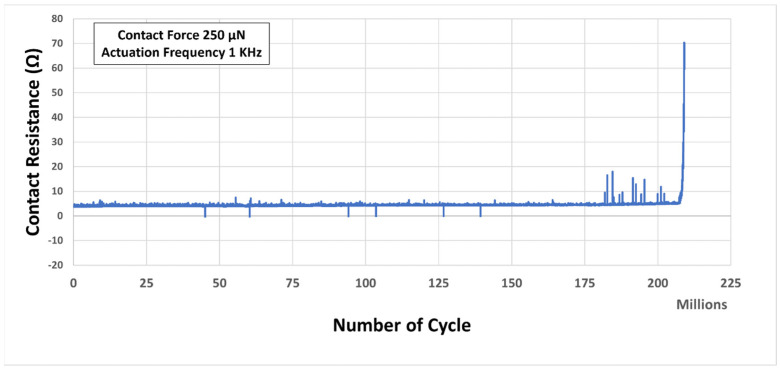
Contact resistance vs. Number of cycles result during cold switched tests (CST).

A direct current (DC) signal was applied to and withdrawn from the contact during CST testing when the contact remained totally closed. Figure 11 shows the CST test result. The test was performed with 250 µN contact force at 1 KHz actuation frequency. The desired number of cycles were gradually raised until the contact failure happened. The result shows that the tested contact reliably and consistently performed for more than 200 million cycles, with an increase of contact resistance from approximately 3.8 Ω to approximately 5.2 Ω. After 181 million cycles, the contact resistance started to fluctuate by giving some higher values. But overall, average contact resistance was within the range mentioned above. The contact resistance increased gradually and rapidly after approximately 207 million cycles. At the end of approximately 209 million cycles, the resistance went up to approximately 70 Ω, which indicates contact failure.

## 5. Conclusions and Future Works

In this work, an improved automated experimental test fixture with high cycle rate and precision was developed and implemented to characterize microcontacts. This fixture provides a much reduced contamination test environment with nm-positioning actuator resolution and nN-range force sensor control. Furthermore, simulation results for beam stiffness, beam resonance frequency, beam actuation frequency, beam displacement, beam restoring force, contact force, contact area, and contact resistance were modelled to better understand microcontact behavior when under load. Based on the design and modeling, the microcontact test structure was fabricated. Moreover, a force sensor and beam performance study were conducted to fully understand initial performance of test fixture and microcontact structure. Initial contact tests (ICT) and cold switched tests (CST) were performed for Au/RuO_2_ bimetallic microcontacts. The test fixture recorded switching operations over millions of cycles, as well as microcontact performance. Future work will include testing microcontacts under various test conditions and contact geometries.

## Data Availability

Data of this study are available within the article.
